# Dissecting the Genetic and Non-Genetic Heterogeneity of Acute Myeloid Leukemia Using Next-Generation Sequencing and In Vivo Models

**DOI:** 10.3390/cancers14092182

**Published:** 2022-04-27

**Authors:** Rhea H. Desai, Niloofar Zandvakili, Stefan K. Bohlander

**Affiliations:** Leukaemia & Blood Cancer Research Unit, Department of Molecular Medicine and Pathology, University of Auckland, Auckland 1023, New Zealand; r.desai@auckland.ac.nz (R.H.D.); n.zandvakili@auckland.ac.nz (N.Z.)

**Keywords:** acute myeloid leukemia, clonal evolution, genetic heterogeneity, epigenetic heterogeneity

## Abstract

**Simple Summary:**

Acute myeloid leukemia (AML) is an extremely aggressive form of blood cancer with high rates of treatment failure. AML arises from the stepwise acquisition of genetic aberrations and is a highly heterogeneous disorder. Recent research has shown that individual AML samples often contain several clones that are defined by a distinct combination of genetic lesions, epigenetic patterns and cell surface marker expression profiles. A better understanding of the clonal dynamics of AML is required to develop novel treatment strategies against this disease. In this review, we discuss the recent developments that have further deepened our understanding of clonal evolution and heterogeneity in AML.

**Abstract:**

Acute myeloid leukemia (AML) is an extremely aggressive and heterogeneous disorder that results from the transformation of hematopoietic stem cells. Although our understanding of the molecular pathology of AML has greatly improved in the last few decades, the overall and relapse free survival rates among AML patients remain quite poor. This is largely due to evolution of the disease and selection of the fittest, treatment-resistant leukemic clones. There is increasing evidence that most AMLs possess a highly complex clonal architecture and individual leukemias are comprised of genetically, phenotypically and epigenetically distinct clones, which are continually evolving. Advances in sequencing technologies as well as studies using murine AML models have provided further insights into the heterogeneity of leukemias. We will review recent advances in the field of genetic and non-genetic heterogeneity in AML.

## 1. Introduction

More than a century ago Charles Darwin proposed the theory of evolution by natural selection of the fittest. In recent years, cancer researchers have accumulated evidence that this theory also applies to the ‘evolution’ of cancers [1,2,3]. During tumor progression, cancer cells continuously acquire genetic changes and the fittest, most proliferative cells are selected for giving rise to distinct tumor subclones. One of the tumor types in which this phenomenon has been studied particularly well is acute myeloid leukemia (AML) [4,5]. AML is a group of highly complex and heterogeneous disorders that arise from the stepwise acquisition of somatic mutations, including chromosomal aberrations, and single nucleotide variants (SNVs), which disrupt the normal mechanisms of self-renewal, proliferation and differentiation in hematopoietic cells [6,7,8,9]. AML is a very aggressive disorder with 5-year survival rates of 30–40% in patients younger than 60 years of age and <10–15% in older patients (60 years and older) [10,11]. The standard of care in AML has remained unchanged since the 1970s and typically consists of a “7 + 3 chemotherapy regimen”, where patients are administered 7 days of cytarabine and 3 days of anthracycline. The “7 + 3 regimen” is given to most AML patients irrespective of their clinical presentation and their cytogenetic and molecular subtype, which is followed by an allogeneic stem cell transplantation in some patients with a high risk of relapse [12]. In the recent past, an increase in the understanding of the pathophysiology of AML has facilitated the development of targeted therapies and more personalized treatment approaches [13,14]. For example, inhibitors of FLT3, IDH1/2 and BCL-2 have been developed and approved by the US FDA for treatment of AML with mutations in these genes or the pathways these genes operate in. However, despite these advances, overall, AML survival rates remain quite poor [14]. In order to improve cure rates in AML and to develop novel subset-specific therapies, it is important to unravel the molecular heterogeneity and genetic landscape of AML. Over the past decade, the advent of next-generation sequencing (NGS) technologies and more recently advances in single-cell sequencing have revealed a very complex genomic landscape in AML, with an AML often consisting of several subclones which have both shared and distinct somatic mutations [7,15,16,17]. In addition to the heterogeneity at the genomic level, AML subclones have been found to exhibit epigenetic, functional and phenotypic heterogeneity, which further adds to the complexity of the disease. 

Animal models have often been used to study the genetic features of human cancers, to characterize mutations as well as to study their contribution to tumor formation, maintenance and progression [18,19,20]. As mice are relatively small, have a well-studied hematopoietic system, well-conserved genes with humans and can be easily genetically manipulated, murine models have been widely used to study AML [20,21]. Transgenic techniques, knock-out/knock-in approaches, xenotransplantations as well as retroviral transduction murine bone marrow (BM) transplantation techniques have been used to model leukemias in mice [21]. Moreover, once a leukemia model is established, cells from a leukemic mouse can be serially transplanted into syngeneic recipients. This enables rapid expansion of the leukemia and thus provides the unique opportunity to study leukemia progression and clonal evolution. Here, we will review the current knowledge on clonal evolution of AML and give a holistic overview of the genetic, epigenetic and phenotypic heterogeneity in AML, with a focus on how mouse models and next generation sequencing studies (NGS) have contributed to our understanding of the disease.

## 2. The Genomic Landscape of AML

AML is a genetically heterogeneous disorder that arises from the malignant transformation of hematopoietic stem cells (HSCs) [22]. Cytogenetic analysis has been used for over three decades to study the genetic basis of AML [23,24]. A number of recurring chromosomal aberrations have been well established as diagnostic and prognostic markers in AML, which include the *AML1/ETO*, *MYH11/CBFB*, *PML/RARA* and MLL fusion genes, to name a few. However, nearly half of all AML patients have a normal karyotype and lack chromosomal abnormalities [6,7]. In the last decade, advances in genomic technologies have resulted in the identification of a plethora of somatic single nucleotide mutations, small insertions, deletions and duplications that contribute to the development of AML. Large scale sequencing studies in patient samples have identified recurrent somatic mutations in more than 200 genes [7,25,26]. The genetic aberrations detected in AML can be broadly grouped based on the functional categories of the genes in which they occur. These include mutations in genes encoding transcription factors (e.g., *RUNX1*, *CEBPA*); those encoding signal transduction proteins such as *FLT3*, *KIT* and the *RAS* family of genes; genes involved in chromatin modification and epigenetic regulation including *DNMT3A*, *IDH1/2*, *TET2* and *ASXL1*; genes of the spliceosome machinery (e.g., *SRSF2*, *SF3B1*, *U2AF1*) and genes encoding members of the cohesin complex (e.g., *SMC3*, *SMC1A*), as well as genes, such as *NPM1*, which is the most frequently mutated gene in AML, that cannot easily be assigned to any of these rather broad functional categories [7,27].

**Figure 1:**
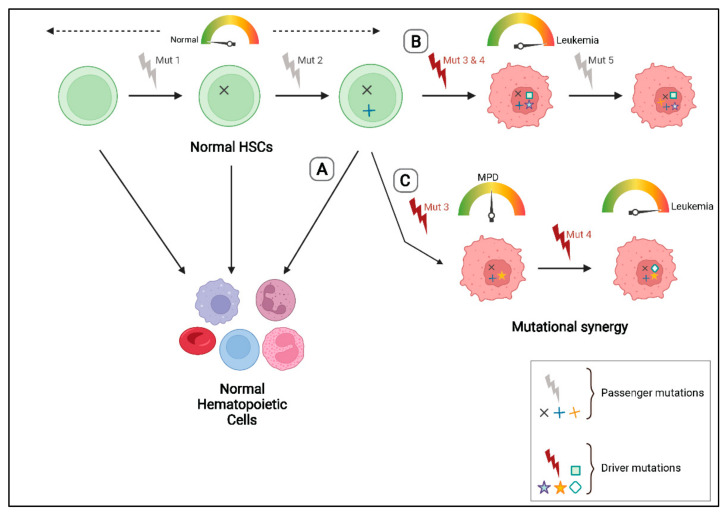
Driver v/s passenger mutations: A normal hematopoietic stem cell (HSC) might acquire passenger mutations that do not impact the growth, proliferation potential or fitness of the cell. (**A**) If a passenger mutation is acquired in the absence of disease-causing driver mutations, the HSC functions normally and can give rise to normal hematopoietic cells of all lineages. (**B**) On the other hand, acquisition of leukemia driver mutations can lead to malignant transformation of the HSC and leukemia development. (**C**) Data from murine models has shown that the introduction of a single strong leukemia driver mutation (e.g., *FLT3* or *NPM1* mutation) often leads to myeloproliferative disorders (MPD) but is not sufficient to lead to AML. The co-operation of other driver mutations is required to initiate leukemia.

Although NGS studies have greatly contributed to elucidating the molecular pathogenesis of leukemias, not all mutations that are identified in a patient sample contribute to malignant transformation. Mutations that provide a proliferative and fitness advantage to a cell and contribute to the disease phenotype are called “driver” mutations, while those that have no effect on the growth and/or fitness of a cell are so-called “passengers” (Figure 1). The “passengers” can be pre-existing mutations that were already present in the hematopoietic cell which subsequently acquired an initial transforming mutation or can be acquired during the course of the disease [27,28,29]. Distinguishing driver mutations from passengers is very challenging. In most cases, a mutation is recognized as a “driver” if it is found to be recurringly mutated in a large cohort of leukemia patients. However, some genes might have a higher probability of being recurringly mutated owing to their size or chromatin organization [27,30]. Therefore, the gold standard for identifying a driver mutation is through functional studies in in vivo models. Murine models have been widely used to study the effect of mutations, for example, those affecting *FLT3*, *NPM1*, *IDH1/2*, and the *RAS* genes, and to study the role of chromosomal translocations in AML [18,19]. 

Murine models have also been an invaluable tool in understanding the contribution of a given genetic change to the development of leukemias. Studies in these models have provided evidence that a single genetic alteration is not sufficient to induce a full-blown leukemic phenotype. Some of the most common mutations detected in patient samples, including *NPM1* or *FLT3,* alone have been shown to lead to myeloproliferative disorders (MPD) but not to AML, in vivo [19,31,32] (Figure 1). However, mice expressing both mutant *FLT3* and *NPM1* or in combination with other known driver mutations develop AML [19,33]. Our group and others have shown that the expression of a single fusion gene alone does not lead to AML. For example, transgenic mice expressing *MLL* fusions or the *CALM/AF10* fusion gene show incomplete penetrance and very long latencies suggesting that cooperating mutations are required for complete malignant transformation [34,35]. We have previously established murine BM transplantation leukemia models (MBMTLM) of *AML1/ETO9a* (a truncated isoform of the *AML1/ETO* fusion) and *CALM/AF10* driven leukemias and shown that the mice acquire additional somatic mutations during the latency period [36,37]. Further, mice genetically engineered to express AML1/ETO (RUNX1/RUNX1T1) have been shown to have a normal life span and do not develop leukemia at all [36,37,38]. This recapitulates observations made in remission samples from patients with the t(8;21) translocation. Miyamoto and colleagues have shown that although *AML1/ETO* was expressed in primitive hematopoietic stem cells (HSCs) of t(8;21) positive patients who had achieved complete remission, these cells were able to differentiate normally and give rise to mature cells of the various blood lineages, thus indicating that the presence of the fusion alone is not sufficient for malignant transformation [39]. Other in vivo studies have reported that *AML1/ETO* when expressed together with mutations in tyrosine kinases such as *FLT3* and c-*KIT* leads to the development of AML [40,41].

From the studies mentioned above and other similar studies, it is now well established that AML is caused by the cooperation of several genetic aberrations that are acquired in a stepwise fashion. According to a hypothesis proposed by Gilliland and Griffin, as few as two cooperating mutations, each belonging to a different class, might be sufficient to induce AML [42,43]. Class I comprises mutations that lead to the aberrant activation of signal transduction pathways and confer a proliferative advantage to the leukemic cells. These include gain-of-function mutations in oncogenes such as *RAS, FLT3-ITD* and *KIT*. The second class of mutations lead to impaired differentiation of hematopoietic stem and progenitor cells and include gene fusions that target transcriptional pathways such as *AML1/ETO*, *CBFβ/MYH11*, and *PML/RARA*, as well as mutations in the transcription factors *RUNX1*, *CEBPA*, and *MLL*, among others [43]. At the time Gilliland and Griffin proposed their model, in the pre-NGS era, our ability to analyze tumor genomes was quite limited. More recently, whole genome and whole exome mutation analyses have uncovered a number of pathogenetically relevant mutations that do not fit into either one of these classes. Moreover, many patients carry no known AML driver mutations, and NGS analyses have not only led to the discovery of new AML associated mutations but also revealed enormous combinatorial diversity and cooperativity between non-recurrent somatic mutations [7,44,45]. Recent studies have also shown that in most cases mutations in genes belonging to different functional categories (e.g., *DNMT3A* and *NPM1*) tend to cooperate with each other, while mutations in genes with similar biological roles (e.g., *TET2* and *IDH2*) are often mutually exclusive [7,46].

The number and kind of somatic mutations found in AML not only differs from patient to patient but can also vary considerably within the same tumor (intra-tumor heterogeneity), which is frequently very obvious when matched samples at diagnosis and relapse are studied. This heterogeneity within a sample is a result of evolutionary processes during the course of the disease. Like most cancers, AML is a clonal disorder, in which an initial transforming mutation leads to a transformed or malignant cell and the descendants of this cell form the founding clone. Individual cells of the founding clone will acquire additional genetic changes leading to the outgrowth of subclones [2,27,47]. During disease progression and maintenance, these clones continually evolve and shape the genomic landscape of the leukemia through the dynamic interplay of emerging new genetic aberrations and intrinsic and extrinsic selective pressures [2,27,47]. Further, individual AML populations may follow distinct models of clonal evolution, which are described below. 

## 3. Patterns of Clonal Evolution

**Figure 2:**
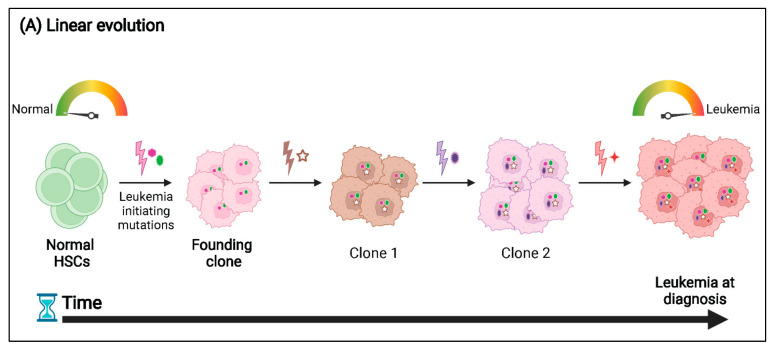

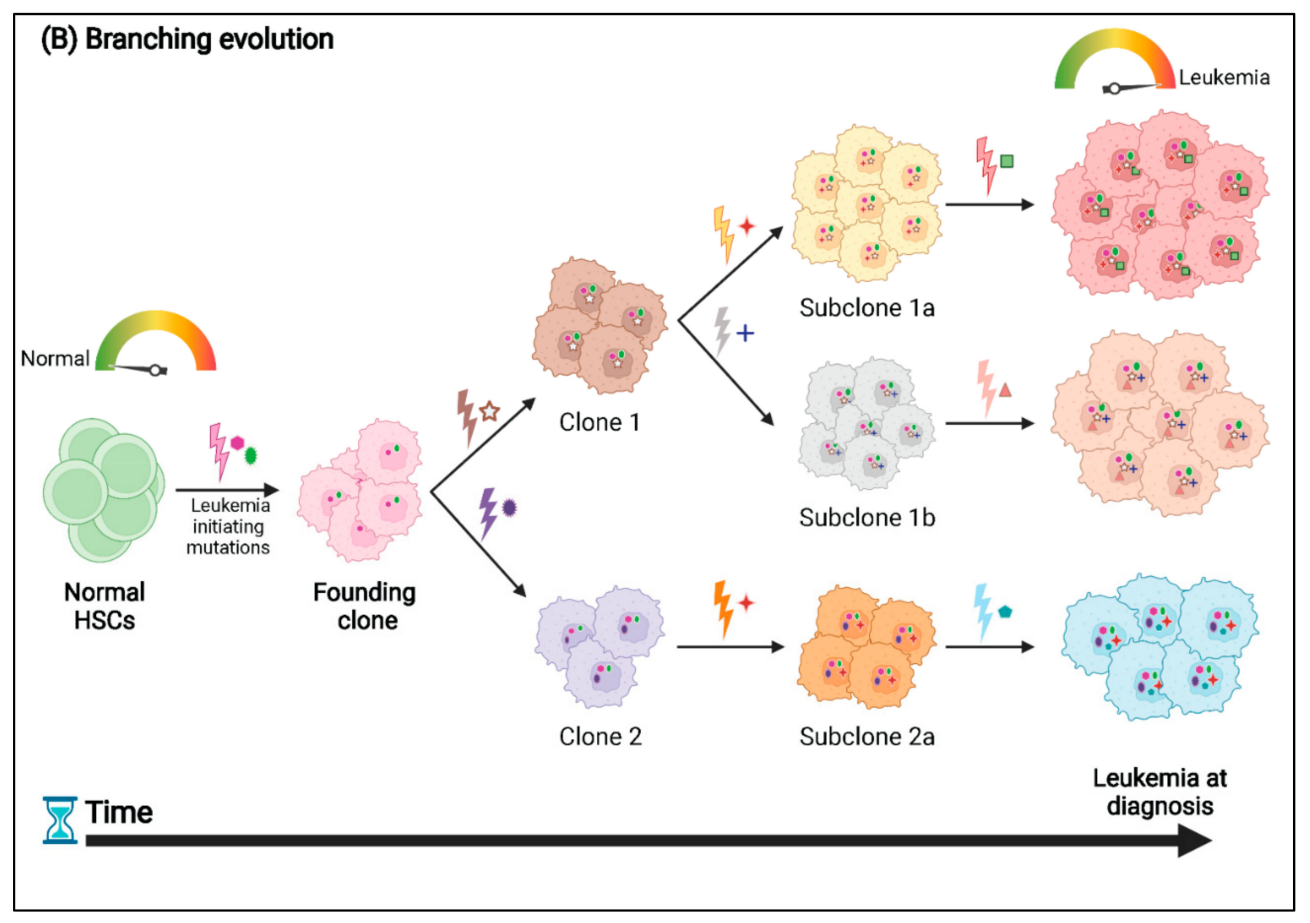
Patterns of clonal evolution: A normal HSC is transformed via the acquisition of leukemia driver mutations, which gives rise to a founding clone. (**A**) Linear evolution: The founding clone might acquire successive driver mutations in a stepwise manner such that each daughter clone contains all the mutations of the parental clone. (**B**) Alternatively, leukemias might follow a branched evolutionary pattern, wherein the founding clone can diverge into several daughter clones, which then evolve in parallel. During branching evolution, two parallelly evolving clones might acquire the same mutation leading to convergent evolution, as depicted in subclones 1a and 2a.

DNA sequencing and mutational profiling of AML samples has revealed two main types of clonal evolution patterns in AML, linear and branching evolution (Figure 2). In linear evolution, new clones arise via the sequential acquisition of new mutations such that each new clone harbors all the mutations of its predecessor clones. During branching evolution, daughter clones diverge from a common parental clone by acquiring distinct mutations. Each daughter clone then evolves in parallel resulting in multiple clonal lineages [2]. Interestingly, an identical mutation may sometimes be observed in two parallelly evolving and related clones during branching evolution (convergent evolution) (Figure 2B). Multiple different mutations in genes belonging to similar functional pathways might also be observed in a branched evolutionary pattern, but these are often present in mutually exclusive clones [48]. In general, leukemias with branching evolutions show a more complex clonal architecture than those that follow a linear trajectory. Both linear and branching evolutionary patterns have been observed during AML relapse, wherein a few AML cells survive therapy and eventually grow out by either acquiring additional mutations that render them resistant to therapy or by losing mutations that are associated with sensitivity to the treatment [2,48].

The clonal complexity and the evolutionary history of a given AML sample is often determined from variant allele fraction (VAF) data obtained from massively parallel DNA sequencing of bulk tumor samples [8]. The VAF is the percentage of reads supporting the mutation divided by the total reads at the mutated position. For instance, Ding et al. performed whole genome sequencing (WGS) on matched leukemia-relapse pairs as well as skin samples (germline tissue) from eight AML patients. Using the VAFs of the mutations, Ding and colleagues were able to estimate the size of the leukemia clones in each AML sample. From their mutation clustering analysis, two major patterns of clonal evolution were detected at relapse, patterns 1 and 2. In cases with pattern 1, the dominant clone at diagnosis gained additional mutations and evolved into the relapse clone, suggesting that either these patients had not been treated adequately (e.g., due to age and/or other factors) or that the dominant clone had mutations that made it resistant to therapy. In cases with pattern 2, some mutations were no longer observed after therapy, suggesting the major subclone had been effectively eliminated. However, the clone at relapse had additional mutations suggesting that a minor subclone at diagnosis had survived treatment, gained additional mutations and expanded at relapse. The authors also reported that irrespective of the pattern of clonal evolution observed at relapse, a dominant mutation cluster representing a “founding clone”, from which all other subclones were derived, was detected in all samples. The founding clone contains mutations that are present in virtually all the tumor cells at presentation and relapse, since the VAF of these mutations is about 40–50% (for heterozygous mutations) [29]. In another study, Grief et al. performed exome sequencing of matched diagnosis, remission and relapse samples from 50 cytogenetically normal AML patients. Based on the mutational patterns observed from diagnosis to relapse, the authors classified the patients into four groups: (i) patients with an identical mutation profile at diagnosis and relapse (“stable”), (ii) patients who gained mutations at relapse (“stable + gain” group), (iii) patients who lost mutations at relapse (“stable + loss”) and (iv) patients who gained and lost mutations at relapse (“mixed” category). Further, the authors found that patients who gained mutations at relapse (“stable + gain” and “mixed” groups) were associated with a significantly longer time to relapse and a favorable prognosis compared to patients who did not gain new mutations at relapse (“stable” and “stable + loss” groups), suggesting that there might be a correlation between clonal evolutionary patterns and time to relapse [49].

Although studies using bulk patient samples have improved our understanding of the evolutionary histories of leukemias, these studies have their limitations as the data from bulk sequencing studies are often not sufficient to accurately reconstruct the clonal architecture and evolutionary patterns of a tumor. In the recent past, advances in single cell sequencing (sc-seq) technologies have enabled the study of mutations in AML with single cell resolution, which has facilitated a better understanding of the clonal architecture of AML [48,50]. In a study by Morita and colleagues, 154 bone marrow samples from 123 AML patients were sequenced using both bulk sequencing and sc-DNA sequencing, the data from which was used to infer the evolutionary histories of the individual samples. The sc-seq analysis provided a definitive picture of the clonal architecture in the patient samples, including evidence for both linear and branched evolution patterns. Moreover, the authors demonstrated that they were not able to elucidate the clonal architecture of most samples of the same cohort of AML patients with the same resolution using the bulk-seq data alone [48].

In addition to sequencing of patient samples at diagnosis and relapse, patient derived xenograft (PDX) murine models are often used to study clonal dynamics of leukemias. In the study by Morita et al., single cells isolated from three AML patients with branching evolutionary patterns were xenotransplanted into immunodeficient mice. Engrafted human CD45^+^ BM cells were then analyzed using sc-seq to study clonal diversity. The study showed that the PDX models closely mimicked the clonal architecture observed in the patient samples. In one of the xenotransplanted samples, 11 of the 12 subclones detected in the original patient sample were also detected in the PDX samples. In the same study, two PDX models were established using samples from another patient who was found to have two AML subclones of similar clonal size. While both subclones had mutations in *DNMT3A*, *ASXL1*, *STAG2*, *BCOR* and *U2AF1*, they had acquired different RAS mutations. One subclone had an *NRAS* mutation, while the other had acquired a mutation in *KRAS*. In both the PDX models, the clone with the *NRAS* mutation was found to expand, while regression of the *KRAS* clone was observed, suggesting that the subclones had different proliferation potentials. Interestingly, similar clonal dynamics were observed in the actual patient on relapse [48]. Sandén et al. established primary (*n* = 57) and secondary (*n* = 27) PDXs using leukemic cells from 26 AML patients. They allowed engraftment and clonal evolution to proceed in the transplanted mice until the first signs of disease were observed. They then performed whole exome sequencing (WES) on the 26 patient samples (collected at diagnosis) and the 84 xenografts to study clonal dynamics of the leukemias. The authors identified five distinct patterns of clonal evolution in the PDXs. These included: “monoclonal leukemias”, which were defined as those samples in which the PDXs harbored the same individual clone detected in the corresponding patient at diagnosis; a “stable” leukemia wherein a dominant clone and a minor subclone were identified in both the patient and the PDX with similar frequencies; the “loss” pattern of evolution where the major clone at diagnosis was lost or detected at a much lower variant allele frequency (VAF) in the PDX models, and the parental clone which contained all but one or two of the AML mutations was found to be retained and was responsible for propagation of the leukemia; the “expansion” pattern wherein a minor subclone detected at diagnosis in the patient sample (average VAFs of 5% at diagnosis) was found to expand and become the dominant clone in the xenografts; and the “burst” pattern where a rare clone at diagnosis expanded in the primary xenografts but was lost in the secondary ones. The “burst” pattern of evolution was detected in 9% of the samples and revealed that the AML cells undergo continuous clonal competition in vivo, and some mutations may confer an initial proliferative advantage leading to expansion of a clone at the expense of long-term self-renewal [51]. More importantly, Sandén et al. showed that in some cases the sample at diagnosis had multiple (sub)clones, including rare clones that could only be detected on serial transplantation and could not be identified on sequencing of the patient sample at diagnosis alone, thus highlighting the significance of PDX models in studying the clonal architectures of leukemias. 

Murine models established using other techniques, such as the MBMTLMs, have also been shown to mimic the clonal evolution patterns detected in AML patient samples. For instance, in MBMTLMs established using two fusion genes, the *CALM/AF10* (minimal fusion) and *AML/ETO9a*, not only were the primary leukemic mice found to acquire several cooperating mutations that were identified via WES, but analysis of successive cohorts of serially transplanted leukemias revealed complex clonal evolution patterns. While in some murine leukemias a founding clone was detected that was found to further evolve on serial transplantation, analysis of other samples revealed a complicated pattern of losses and gains of mutations suggesting the expansion and contractions of leukemic subclones [36,52]. Interestingly, the murine leukemias analyzed in the study recapitulated some of the patterns of clonal evolution observed in human AML patients that had been treated and then relapsed, even though no extrinsic selective pressures (e.g., chemotherapy) were applied [36,52]. These studies demonstrate that murine models provide a useful resource to study clonal evolution patterns in AML.

## 4. Mutational Order and Pre-Leukemic HSCs

Despite the complex evolutionary patterns that have been identified in AML samples, recent studies suggest that mutations are most likely acquired in a specific order and the order in which mutations are acquired impacts the phenotype and clinical response of AML. Several studies have shown that mutations in genes encoding epigenetic regulators such as *DNMT3A, IDH1/2*, *TET2* and *ASXL1* are predominantly acquired early on, during initiation of the disease. These mutations are frequently found at high VAFs indicating that they are present in virtually all tumor cells in a sample and represent the “founding clone” [29,48,49,53,54]. On the other hand, mutations in signal transduction genes such as *FLT3* and *RAS* are found to occur later during disease progression. Consistent with these findings, sequencing of matched sample pairs at diagnosis and relapse has revealed that mutations in epigenetic modifiers are often retained after therapy and have also been found to persist in patients in complete remission, while mutations in *FLT3* and *RAS* tend to be unstable and either expand or are lost in the relapse sample indicating that these mutations are subclonal [49,55,56]. In addition, although mutations in *NPM1*, one of the most frequently mutated genes in AML, are often thought to be disease initiating mutations, Shlush et al. demonstrated that in AML samples containing *DNMT3A* and *NPM1c* mutations, the *DNMT3A* mutation preceded the mutation in *NPM1* [54]. 

**Figure 3:**
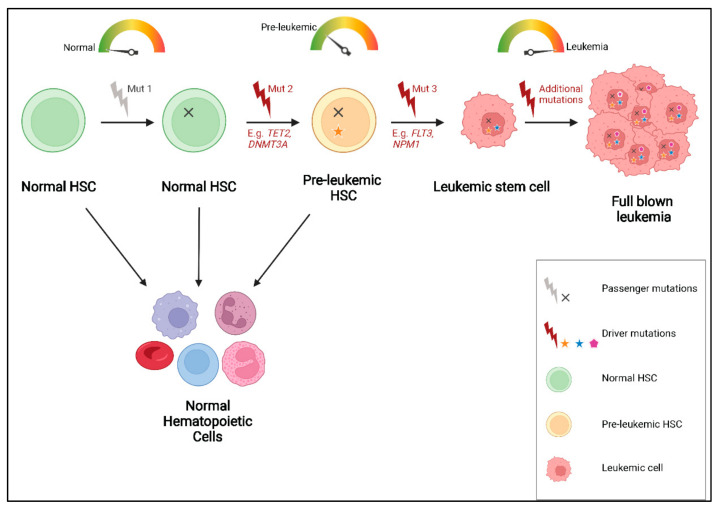
Pre-leukemic HSCs and CHIP/ARCH: Some healthy individuals, particularly older people, harbor mutations in genes such as *TET2*, *DNMT3A* or *IDH1/2* (mutation 2), resulting in a population of pre-leukemic HSCs, which retain the ability to proliferate and differentiate into normal blood cells. However, on acquiring additional leukemia driver mutations (mutation 3), these cells might undergo malignant transformation which can eventually lead to leukemia development.

Intriguingly, recurring mutations in genes involved in epigenetic regulation, particularly in *DNMT3A*, *TET2* and *ASXL1* are not only detected in patients with hematopoietic malignancies, but have also been recurrently identified in samples from healthy people, especially in those obtained from elderly individuals [57,58,59]. These mutations accumulate in normal HSPCs, referred to as pre-leukemic HSCs, which have been shown to retain the ability to regenerate the entire hematopoietic hierarchy while possessing a competitive repopulation advantage over non-mutated HSCs in xenotransplantation assays [54]. This phenomenon, called clonal hematopoiesis of indeterminate potential (CHIP) or age-related clonal hematopoiesis (ARCH), is defined by the presence of a known hematologic-associated mutation with a VAF of at least 2% in an otherwise healthy individual [54,59]. The incidence of CHIP/ARCH increases with age and a subset of these cases acquire additional cooperating mutations and may eventually progress to overt hematologic malignancy, including AML [58] (Figure 3). This further explains why these mutations are often found to be present in the founding clone in AML and persist during remission and relapse. 

To gain a better understanding of CHIP, Loberg et al. developed an in vivo dual recombinase system by combining interferon-inducible Cre and tamoxifen-inducible flippase recombinases, which enabled the sequential induction of a *Dnmt3a* mutation (corresponding to the R882H hotspot mutation in humans) and a *Npm1* mutation (corresponding to the *NPM1c* mutation), respectively. The study showed that mice that were first induced for the *Dnmt3A* mutation followed by the *Npm1c* mutation developed MPD, which progressed to AML on subsequent transplantations. Notably, increasing the time between induction of the *Dnmt3a* mutation and that of the *Npmc1* mutation resulted in a more aggressive MPD and a decreased overall survival in the mice suggesting that when the *Dnmt3a* mutant clone was allowed to expand for a longer duration, it increased the risk of progression to malignancy [60]. This study highlights that the order and time at which mutations are acquired may indeed have an impact on the clinical presentation of the leukemia. Although several studies have now clearly established a role of CHIP in inducing hematological malignancies, a number of key questions remain unanswered. For instance, the precise mechanisms that mediate the clonal expansion of cells harboring these mutations as well as the duration of the pre-leukemic phase of AML and its implications on the disease are not fully understood. Establishing murine models that recapitulate CHIP may lead to a better understanding of the underlying mechanisms of this phenomenon, to the development of better therapeutic strategies targeting pre-leukemic HSCs and potentially provide the opportunity to intervene before the onset of AML.

## 5. Phenotypic-Genotypic Correlation in AML

Leukemic cells are not only characterized by heterogeneity of their genetic lesions, but also show distinct phenotypes, including heterogeneous cell morphology and surface marker expression. A number of studies have demonstrated that phenotypic heterogeneity arises as a result of heterogeneity at the genomic level. For example, Todisco et al. have shown that the co-occurrence of a mutation in the *SRSF2* gene (*SRSF2*^P95^), which encodes a splicing factor, and other driver genes can be correlated with distinct clinical phenotypes. When the *SRSF2*^P95^ mutation co-occurs with mutations in *JAK2* or *MPL* myelofibrosis is observed. On the other hand, co-occurring mutations in *TET2* and the RAS pathway genes lead to monocytosis and leukocytosis, respectively, while co-occurring mutations in *STAG2*, *RUNX1* or *IDH1/2* correlate with a blastic phenotype. Therefore, the diverse phenotypes observed in different patients with *SRSF2*^P95^-mutated neoplasms can be attributed to inter-patient mutational heterogeneity [61]. In another study, Jiang et al. found three cell populations in t(8;21) AML patients. These included a “CD34^+^CD117^dim^” population that was positive for the HSPC marker CD34 and had low expression of CD117 (or c-Kit, another HSPC marker), a “CD34^+^CD117^bright (bri)^” population which had high expression of CD117, and a population of “abnormal myeloid cells with partial maturation (AM)”. These compartments showed heterogeneous biological features such as variability in gene expression, morphology, proliferation ability and response to therapy. Interestingly, the CD34^+^CD117^bri^ cells were less susceptible to chemotherapy than the other two populations. In addition, patients with a higher level of the CD34^+^CD117^bri^ cell population showed worse overall and relapse-free survival than patients with fewer CD34^+^CD117^bri^ cells. This suggested that a higher proportion of CD34^+^CD117^bri^ cells in t(8;21) AML patients might be associated with a poor prognosis and inferior outcomes [62]. Overall, this study demonstrated that patients with the same type of leukemia might harbor different leukemic cell populations with heterogeneous features and clinical outcomes.

Not only inter-patient but also intra-patient phenotypic heterogeneity is frequently observed in leukemia. In some patient samples, the pattern of the cell surface antigen expression profile of the leukemic cells has been shown to evolve from diagnosis to relapse [63,64,65,66,67,68]. For example, antigen expression analysis of leukemic samples from 136 AML patients showed a change in immunophenotype in 91% of the patients from diagnosis to relapse. The most frequent changes observed at relapse were increased expression of CD13 (predominantly expressed on myeloid cells), CD33 (an immature myeloid cell marker) and CD34 (found on stem and progenitor cells) and a decrease or loss of expression of CD56 (typically expressed on natural killer cells, but also other immune cells), CD19 (a B-cell marker) and CD14 (expressed on monocytes, macrophages and some granulocytes) [64]. Similarly, in another study of 47 patients with refractory (*n* = 22) and relapsed AML (*n* = 25), the immunophenotype of the leukemic cells was found to change in the majority of the patients during the disease course. The most frequent changes observed were in the expression of CD13, CD33, CD56, CD7 (an early T-cell marker), CD4 (mainly expressed on helper T-cells) and CD11b (a myeloid marker) [63]. The authors also reported cytogenetic clonal evolution in 14% of the refractory and 44% of the relapsed AML cases. They suggested that cytogenetic clonal evolution could partially explain the immunophenotypic shift observed in the study. However, other factors like clonal selection in response to therapy could also contribute to the change in immunophenotype [63].

The phenotypic shift in leukemia might result from the acquisition of new mutations and evolution of a pre-existing (sub)clone, from the emergence of a new clone at relapse, or the outgrowth of a minor (sub)clone that was present in the sample at diagnosis, which was resistant to therapy and expanded to drive relapse [67]. Although the correlation between clonal architecture and phenotypic heterogeneity is not entirely understood, studies have shown that cells from genetically distinct subclones of the same tumor may have distinct immunophenotypes. In a study by Klco and colleagues, whole genome and targeted sequencing of unfractionated leukemic cells from the BM and the peripheral blood (PB) of patients with *de novo* AML revealed that different subclones of the same tumor gave rise to leukemias with distinct morphologies and phenotypes [47]. For example, one of the samples analyzed was an acute monocytic leukemia with predominantly monocytic features and about 3% blasts in the PB (at diagnosis). On analysis of the clonal architecture of this leukemia, using matched diagnosis and relapse samples, three subclones (subclones 1, 2 and 3) were detected. Subclones 1 and 2 were detected at diagnosis but not in the relapse sample, whereas subclone 3 (a rare subclone at diagnosis) was found to expand and became the dominant subclone at relapse. Interestingly, the relapsed leukemia showed blastic characteristics instead of the monocytic morphology observed at diagnosis. On performing targeted sequencing on purified myeloid blasts (isolated from the sample at diagnosis), the authors found that the blasts had the somatic variants linked to subclone 3, which had become the dominant clone at relapse [47]. Similarly, analysis of myeloid blasts and monocytes from another patient sample with acute myelomonocytic leukemia revealed that different subclones had distinct morphologies [47]. These results demonstrated that distinct genetic subclones within an individual leukemia can be associated with different morphologic or immunophenotypic features.

Additionally, in a comprehensive study of 50 AML patients, de Boer et al. demonstrated that subclones sorted based on plasma membrane protein expression from each individual patient were genetically distinct. For example, in one patient, they identified two subclones with a mutation in *NRAS* and *WT1*, respectively, both of which evolved from a founding clone with a mutation in the *CEBPA* gene. The two subclones showed different cell surface marker expression profiles. The *NRAS* subclone was IL1RAP^+^ (i.e., interleukin 1 receptor accessory protein positive), whereas the *WT1* subclone was IL1RAP^−^. In another patient, they found a subclone with a *FLT3*-ITD mutation, which had evolved from a founding clone with mutations in *DNMT3A* and *RUNX1*. Interestingly, the *FLT3*-ITD mutation was found to correlate with expression of CD25 (an activated T-cell marker) [68]. The correlation between *FLT3*-ITD mutations and CD25 expression has also been reported in other studies [69]. 

Furthermore, using simultaneous profiling of single-cell DNA and cell surface protein expression (scDNA + protein-seq), Morita et al. showed an interesting association between the mutational pattern and immunophenotypes in 26 AML patients. Their results indicated that mutations in the *NPM1* or *IDH1/2* genes correlated with reduced expression of the HLA-DR (human leukocyte antigen–DR isotype) and CD34 markers on leukemic cells. In contrast, *TP53* mutations were linked to CD34 overexpression. Using these data, they also found a correlation between genetic and phenotypic evolution in their AML samples. For example, in one of the samples with a linear evolutionary pattern, they found that the stepwise acquisition of mutations in *TET2*, *U2AF1*, *DNMT3A* and *NRAS* was associated with phenotypic alterations. In this patient, a single mutation in *TET2* correlated with the expression of both lymphoid (CD3, CD19 and CD22) and myeloid (CD11b) markers, indicating that the *TET2* mutation was most likely acquired in a pre-leukemic HSC. *TET2-U2AF1* double-mutated cells had the expression of the same markers but at a lower level, as well as expression of early myeloid markers (CD123 and CD13). Then, on acquiring a *DNMT3A* mutation (*TET2-U2AF1-DNMT3A* triple-mutated cells), the cells were found to express the hematopoietic stem cell markers CD34 and CD117. Finally, after acquisition of a mutation in *NRAS*, the quadruple-mutated cells showed a myeloblastic phenotype (CD33^+^, CD34^+^ and CD38^+^), similar to the phenotype of the leukemic blasts [48]. 

**Figure 4:**
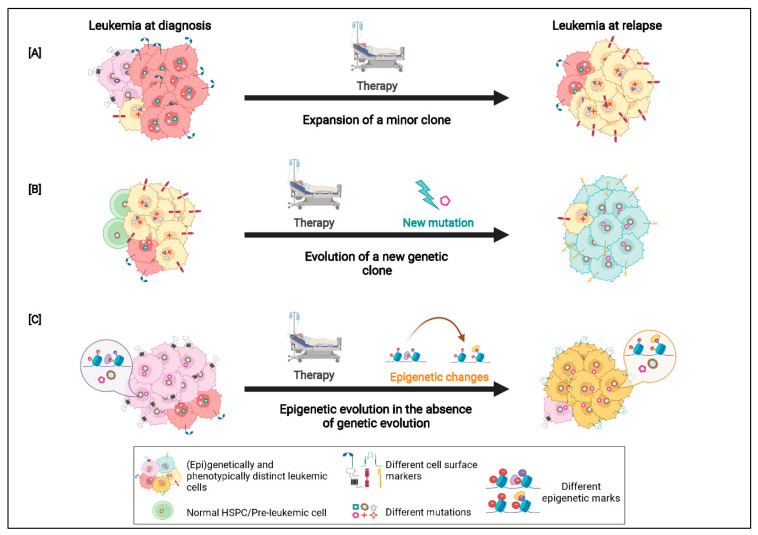
Genetic, phenotypic and epigenetic evolution in AML: (**A**,**B**) On applying a selective extrinsic pressure (e.g., chemotherapy), the leukemia undergoes genetic evolution. The dominant clone at diagnosis may be lost or considerably reduced in the relapse sample, with either a minor clone at diagnosis expanding at relapse (**A**) or, due to the acquisition of new mutations, a new subclone might appear at relapse (**B**). In both instances, the new dominant clone at relapse may have a different cell surface expression profile compared to the major clone at diagnosis, thus resulting in an immunophenotypic shift of the leukemia. (**C**) In some cases, a change in the leukemic phenotype is observed between samples at diagnosis and relapse, although no changes in the genetic clonal composition is observed. There is now evidence that in such cases changes in the epigenomic landscape of the leukemia might be responsible for driving disease progression and relapse.

Similar observations with regard to genotypic-phenotypic evolution have also been made in murine models of AML. In a *CALM/AF10* driven MBMTLM established by our group, limiting dilution assays (LDAs) were performed and leukemias established from single leukemia stem cells (LSCs; reviewed in [22]) were characterized in detail. WES and immunophenotypic analysis of the murine leukemias revealed a correlation between genetic and phenotypic heterogeneity [70]. The primary *CALM/AF10* leukemia established in the study was found to have a predominantly myeloid phenotype with only 4% of its cells expressing the B-cell marker B220. WES analysis of this leukemia revealed the presence of additional somatic mutations. From the WES analysis, three distinct genetically defined clones were identified in the primary leukemia including a founding clone, subclone 1 and subclone 2. On performing serial transplantations with 50,000 leukemic cells from the primary and a secondary leukemic mouse, expansion of subclone 1 and a reduction in the size of subclone 2 was observed, with subclone 2 being lost in the tertiary transplants. Interestingly, an increase in the proportion of the B220 positive cells was observed in the tertiary bulk transplanted murine leukemias, but not in the secondary leukemias. We then performed LDAs using cells from one of the secondary leukemias and found that the tertiary recipients had greatly varying B220 marker expression, ranging from 2 to 85%. Four of these tertiary leukemias had each originated from a single LSC, isolated from the same secondary donor leukemia, and were characterized at the genomic level using WES. One of these leukemias had a B220 expression profile similar to that of the bulk transplanted tertiary samples and also showed an identical genomic profile, with subclone 1 expanding and loss of subclone 2. Another leukemia that arose from a single LSC was found to have very low B220 expression (2%) which correlated with the acquisition of a new subclone (subclone 3) and loss of subclones 1 and 2. The remaining two tertiary leukemias that were sequenced, each of which had originated from a single LSC, had high B220 expression and a much longer latency compared to the other leukemias, which correlated with an expansion in subclone 2 and loss of subclone 1. Surprisingly, an expansion of subclone 2 was observed only in these two samples, while in all other tertiary samples subclone 2 was lost [70]. These results demonstrate that cells belonging to distinct genetically defined subclones within the same leukemia can show striking differences in immunophenotype. This study also showed that by transplanting single LSCs into recipient mice, it is possible to unravel the complex genomic architecture of the leukemia, which might not be apparent from serial bulk transplantation assays. Collectively, the results from the various studies described here indicate that leukemias are not only genetically diverse, but the distinct combination of somatic mutations present in an individual clone also leads to phenotypic variations within the leukemia (Figure 4).

In addition to providing insights on the complex evolutionary patterns, a probable mutational order and an extremely dynamic genetic and phenotypic landscape in AML, sequencing studies have also revealed that the overall mutational burden is relatively low in AML compared to other cancers [27,71]. Further, in some AML cases, patients do not show clonal genetic diversity upon relapse and yet exhibit resistance to chemotherapy [72,73]. This led to the hypothesis that other mechanisms, such as epigenetic changes, may also contribute to leukemia evolution and relapse. Indeed, epigenetic alterations are now emerging as one of the key players in the pathogenesis of AML.

## 6. The Epigenetic Landscape of AML

Epigenetic modifications are chemical changes to the DNA molecule or the chromatin proteins, which do not change the DNA sequence but influence gene expression, and include DNA methylation, histone modifications such as methylation, acetylation, phosphorylation and ubiquitinylation affecting chromatin remodeling. Epigenetic modifications can be stably transmitted through mitosis. However, unlike genetic aberrations, epigenetic marks show great plasticity and can change during normal development, during the course of a disease and in response to external stimuli. This often leads to epigenetic heterogeneity, which can be defined as genetically identical cells exhibiting distinct epigenetic states, leading to transcriptional diversity within a clonal population of cells [74,75]. Along with heterogeneity at the genomic level, it is now becoming evident that cancers in general, including AML, display epigenomic diversity. Further, epigenetic plasticity allows tumor cells to extensively explore various epigenetic states and discover those that provide a proliferative and survival advantage [74,76]. 

As mentioned in previous sections, mutations in genes encoding epigenetic modifiers are frequently found in AML and are also detected in pre-leukemic HSCs. The most common epigenetic mutations have been detected in genes involved in DNA methylation, which include *DNMT3A*, *TET2* and *IDH1/2*, as well those involved in histone modifications such as *ASXL1* and *EZH2*. The dysregulation of these genes leads to aberrant methylation or other epigenetic patterns in tumor cells. Somatic mutations in *TET2* or in the isocitrate dehydrogenase enzymes, *IDH1/2*, are often associated with hypermethylation signatures and subsequent inactivation of target genes that result in impaired hematopoietic differentiation. On the other hand, AML cases with mutations in the DNA methyltransferase enzyme, *DNMT3A*, have been found to exhibit hypomethylation and thereby activation of target genes. Mouse models of mutant *IDH1/2*, *TET2* and *DNMT3A* recapitulate the epigenetic features observed in AML patients with these mutations, supporting their functional significance [77,78]. In addition, mutations in other genes have also been linked to distinct DNA methylation profiles [7,77]. Samples with *CEBPA* mutations, as well as those with the *PML/RARA*, *AML1/ETO* and *MYH11/CBFB* fusions, show specific patterns of methylation losses and gains compared to normal CD34^+^ BM cells [7,76,77]. Studies have also reported that cooperation between somatic mutations such as *FLT3* and *TET2* or *NPM1* leads to an increase in epigenetic diversity in AML cells, and an enhanced epigenetic diversity has been associated with inferior patient outcomes [72,76,79].

Interestingly, there is a growing body of evidence that suggests that epigenetic changes may also drive disease progression and relapse in AML, independent of underlying mutations (Figure 4C). This has been demonstrated in a study by Li et al., wherein they performed a comparative analysis of cytosine methylation patterns at selected loci along with mutational analysis of paired AML patient samples at various time points. The authors reported changes in methylation patterns between matched sample pairs at diagnosis and relapse. The magnitude of change between patients was found to be highly variable and independent of factors such as patients’ age, AML subtype or the number and type of somatic mutations. Notably, on comparing the genetic and epigenetic profile of each patient they found that in some patients, although the genetic clonal composition remained stable during disease progression, there were considerable changes in the DNA methylation patterns, indicating that epigenetic and genetic diversification do not always follow the same kinetics during leukemic progression and can be, to an extent, independent of each other [72]. In a recent study, Nuno et al. performed an assay for transposase-accessible chromatin with high-throughput sequencing (ATAC-Seq) as well as targeted gene sequencing using a myeloid malignancy panel on paired diagnosis and relapse AML samples. Several samples were found to exhibit the same mutations at diagnosis and relapse. ATAC-seq analysis of these samples revealed substantial epigenetic evolution, with purified AML blasts displaying a more differentiated myeloid cell profile at diagnosis, which was found to shift to a more primitive stem and progenitor-like pattern at relapse. The shift in the epigenetic profile correlated with a loss of accessibility of *PU.1* and *CEBPA* transcription factor motifs and an increase in accessibility of the *GATA* and *RUNX* motifs [73,80]. PU.1 and CEBPA are transcription factors required for myeloid differentiation, while GATA and RUNX1 are transcription factors that function in more immature cells. This work shows that epigenetic remodeling may contribute to leukemic progression and relapse in the absence of genetic evolution. 

Alterations in DNA methylation patterns have also been associated with leukemic progression in AML murine models. Sonnet et al. examined the epigenetic profile of a mouse leukemia model driven by the hypomorphic expression of the PU.1 transcription factor [81]. The study described the methylation patterns observed in the murine BM cells at three distinct disease stages, defined based on blast counts: pre-leukemic (<20% BM blasts), early leukemic (20–50% BM blasts) and late leukemic (>50% BM blasts). While aberrant DNA methylation was detected at all three stages of the disease, epigenetic diversity was found to increase with progression of the leukemia, with the late-leukemic stage correlating with an increase in the number of hypermethylated regions. They also identified a number of AML-associated genes, including *CEBPA*, that were aberrantly methylated at the pre-leukemic stage, indicating that the altered epigenetic state of these genes may play a role in the onset of AML [81]. Hypermethylation at the *CEBPA* locus has also been reported in human AML and has been proposed as a useful prognostic marker for AML patients [82]. 

The studies described above strongly suggest that epigenetic heterogeneity and plasticity are key drivers of leukemic transformation and disease progression, particularly during relapse. However, the mechanisms that mediate epigenetic heterogeneity and the complex interplay between genetic and epigenetic signals are not completely understood yet. The study by Sonnet and colleagues showed that mouse models of leukemogenesis can also be utilized to identify altered epigenetic states and study epigenetic evolution during leukemia progression. With advances in technologies such as ATAC-seq, it has now become possible to perform epigenetic analysis combined with genetic and transcriptomic analysis not only on patient samples but also in murine models. Such multi-omic studies of murine leukemia models at different stages of leukemia will further help us to understand the role of epigenetic reprogramming in the initiation and progression of AML. 

## 7. Conclusions and Future Implications for Research

AML is a highly heterogeneous and aggressive malignant disease originating from hematopoietic stem cells. Although our understanding of the molecular basis of AML has improved greatly in the last four decades, overall survival rates, especially for older patients (>60 years of age), remain quite poor. Even though young patients initially respond to therapy and achieve complete remission, a majority of these patients eventually relapse [10,83]. In order to improve current treatment strategies and to develop new ones, we first need to gain a better understanding of the pathogenesis of AML and answer some key questions, including why some patients develop resistance to therapy and why patients with a similar clinical presentation show variable drug responses. 

As technologies (summarized in Table 1) that allow us to perform in-depth molecular characterization of cancers have become relatively affordable and thus easily accessible, our understanding of the underlying heterogeneity of AML has further deepened. Numerous studies, some of which are described above, have explored the genetic, epigenetic, transcriptomic and phenotypic profiles of AML samples. In addition, there is an increasing interest in the proteomic and metabolomic signatures in AML and their correlation with distinct genomic profiles (research in this field is in its nascent stages and beyond the scope of this review) [84,85,86]. A common theme emerging from these studies is that AML is more complex and heterogeneous than was previously thought. The subpopulations present within a single AML sample can not only be genetically and thereby phenotypically distinct but can also show epigenetic variations, and these changes in the genome and epigenome together appear to be driving the onset, progression and evolution of AML. 

In addition to sequencing studies of human AML samples, murine models have been an invaluable tool in the study of AML. While NGS studies of patient samples have provided us with a wealth of knowledge on various aspects of cancers, murine models have been essential for validating the contribution of different genetic and epigenetic aberrations to the development and progression of a cancer, particularly of AML, as well as for studying the clonal evolution of AML. As described above, in some cases it is possible to truly unravel the genomic landscape of a given AML patient sample only by performing xenotransplantation assays and establishing at least a few PDXs from each sample. Several groups have demonstrated that by performing serial transplantations in murine leukemia models, it is possible to recapitulate the key features of human AMLs. Thus, future research on clonal evolution and heterogeneity in AML should focus on an in-depth molecular characterization (multi-omic studies) of patient samples as well as murine leukemia models. Well characterized murine models will not only provide us with a deeper understanding on the underlying molecular pathogenesis of AML but can also be utilized to test novel drugs and treatment regimens in pre-clinical settings. 

## Figures and Tables

**Table 1:** An overview of the frequently used NGS technologies that have provided insights into the complex genetic, transcriptomic and epigenetic landscapes of AML (for a comprehensive review, refer to [87]). These techniques are used for the sequencing of bulk AML samples as well as for single-cell sequencing, depending on the aim of the study.

Sequencing Technology	Description
**Genetic Analysis**	
Whole genome sequencing	Identifies the complete range of genomic alterations, including point mutations, indels, copy number changes as well as structural chromosomal rearrangements
Whole exome sequencing	Reveals changes in the coding region of the genome
Targeted or gene panel sequencing	Deep sequencing of a panel of genes that are recurringly mutated or have a prognostic significance in AML
**Gene expression analysis**	
mRNA sequencing	Identifies alternative transcripts, gene fusions and allele-specific expression patterns in the coding transcriptome
Whole transcriptome analysis	Detects changes in expression of both coding and noncoding RNA
**Epigenetic analysis**	
Methyl sequencing	For studying changes in methylation patterns at a single nucleotide level, mainly using bisulfite treatment. Several methyl-seq. strategies have been developed including whole genome bisulfite sequencing and targeted approaches as well as reduced representation bisulfite sequencing, which enriches for CpG islands
ChIP-seq analysis	Combines chromatin immunoprecipitation with NGS for the identification of binding sites of DNA-associated proteins across the genome. Used to map histone modifications and transcription factor binding sites.
ATAC-seq analysis	Used for determining regions of chromatin accessibility and to map DNA binding proteins for the identification of active promoters, enhancers and *cis*-regulatory elements.
